# Observations upon the Nature and Treatment of Parotitis Mercurialis

**Published:** 1831

**Authors:** George R. Morton

**Affiliations:** Coshocton, Ohio


### WESTERN JOURNAL

OF THE

### MEDICAL AND PHYSICAL SCIENCES.

OCTOBER, NOVEMBER, AND DECEMBER, 1832.

### Essays and Cases.

---

**Art. I.** *Observations upon the Nature and Treatment of Parotitis Mercurialis, by* George R. Morton, M. D. *of Coshocton, Ohio.*

In the wide range of research and discovery, which Pathological science has opened to its professors, the glandular system, in its anatomical structure, its diseases, and their remediate management, has attracted a large share of the attention of medical writers, especially of the present day. The subject has been investigated with the keenest discrimination, by men whose names stand high in our esteem, and whose talents have elicited a fund of knowledge of the most practical usefulness.—The mysteries with which early pathologists have enveloped it, are fast disappearing; and a theory more consonant with its important functions has arisen, which bids fair soon to reach perfection. But great as have been the discoveries, and essential as are the alterations which these have introduced into the treatment of glandular disorders, much yet remains unknown and uninvestigated, or at best but imperfectly noticed and understood. It is thus with the disease in question. We have searched over the works on medicine, both ancient and modern, and have looked in vain for any author, whose writings would lead us to infer even the slightest acquaintance with it. If they have described, accidentally, any disease at all approximating to it, they have noticed it as a symptom sometimes consequent upon, and connected with "malignant and pestilential fevers;" and have stumbled over it, like Clio of old, without the least idea of its true character and interesting nature. None, however, have noticed it as a distinct and peculiar disease, *sui generis*, and as such deserving attention. As such, we intend to describe it; not as a *new* disease, but as one whose rare occurrence has rendered it unnoticed, notwithstanding its singular features, and terrible fatality. In endeavouring to introduce it to the notice of the medical profession in general, we are not anxious to arrogate to ourselves the palm of originality—but are willing and proud to rank ourselves as subalterns in the task. To our friend Washington L. Atlee, M. D., of Lancaster, Pa., belongs the honor of having first collected and arranged whatever information we may, in the following pages, present, in an inaugural Thesis, laid before the Faculty of Jefferson Medical College, in 1828. Though we differ from him in some particulars, which at a future day we may notice, yet we embrace with pleasure this occasion of testifying our sincere respect for his sterling talents and laborious research.

Nosology is now so indispensably a part of the science of medicine, both from its convenience and utility, that we are somewhat bound, as true disciples of Hippocrates, to give our subject "a local habitation and a name" among the long list of disorders to which "flesh is heir." We would, therefore, designate it by the title of *Parotitis Mercurialis*—and define it as a specific, distinct, and peculiar inflammation, originating in, and primarily affecting the parotid gland; and which, if suffered to run an undisturbed course, speedily ends in gangrene, mortification, and death. The distinctive term *mercurialis*, as our knowledge of the disease progresses, may be considered objectionable; but in the present situation, we are disposed to prefer it to any other we can at this time suggest.

*Symptoms.* Our present limited acquaintance with this truly appalling and singular inflammation, and the paucity and unfrequency of cases, debar us from giving so full and distinct a detail of the attendant symptoms, as we could desire. We will, therefore, confine ourselves to a sketch of the most prominent, and such as cannot be easily mistaken by an observant eye, especially when considered in connection with one another. From the few cases which have fallen under our own observation, or have been communicated to us by our friends, we have never known parotitis mercurialis to exist as a primary, independent disorder, or under any other circumstance than as a sequent of other diseases, particularly *febrile*, where *mercury* has been exhibited in the process of cure. When, therefore, in cases of febrile or other disorders, (where mercury has been administered,) arrived at that stage where the patient is just lingering between disease and convalescence, he complains suddenly of a sense of swelling beneath and in front of the ear, extremely tender upon the slightest pressure, either of the finger or of the pillow, rapidly increasing into a circumscribed tumour, which is hard, painfully lancinating, and spreading anteriorly, with pain on every motion of the jaw, we may, without hesitation, set it down as a case of genuine parotitis mercurialis. The enlargement of the parts is exceedingly rapid in its advancement, and speedily involves the surrounding structure in its disease. Extending, in a few hours, over the whole size of the head, affecting the palpetræ, the base and angle of the lower jaw, and the side of the neck; it embraces in its fearful progress, the connecting cellular tissues of the neighboring parts—the periosteum, the muscles, the parenchymatous substance of the gland itself, until the whole is one agglutinated mass of inflamed and diseased matter. The skin over the tumor is red, smooth, and glossy; and, when felt by the hand, emits a heat which conveys the sensation of being pricked by needles, to the experimenter. The tension of the skin is very great, and the swelling resembles, in appearance and colour, at least in some degree, an inflamed spleen, covered by a serous membrane. As the disease advances, the pain becomes excruciating, of a lancinating, burning character: the process of deglutition becomes difficult and painful: the voice is paraphoniac: the countenance is flushed and livid, and the sense of strangulation is oppressive. The tumescence having reached its acme, the pain most usually subsides, or is not complained of: the head is affected with a dull, heavy feel: the tumour itself becomes livid: deafness of the ear of the affected side; and finally, a low, muttering delirium, with other symptoms of an oppressed brain, and a sinking condition of the system, supervene. The tumour now becomes somewhat soft, and fluctuation is perceptible: a gangrenous suppuration ensues: the abscess bursts and discharges, generally in small quantities, a sero-purulent matter. Contrary to what might be expected, no mitigation of the symptoms appear: the central or glandular portion of the tumour still continues firm and hard: sloughing of the cellular tissue, resembling wet tow, takes place: and the pulse, which, in the beginning, was hard, quick, and contracted, is now small, weak, and exceedingly rapid, beating as high as 150 or 160 in a minute. The disturbed condition of the cerebral system, instead of abating is increasing, and the patient sinks from exhaustion and debility; or, in case of the abscess bursting inwardly, from strangulation. If undisturbed, the disease generally runs its course in four or five days.

Such are a few of the most prominent and characteristic features of this terrific malady, which a cursory and limited attention to the subject has enabled us to present.

We have never known it to occur during the continuance of an active ptyalism; but only when the constitutional effects of mercury had, in a great measure, subsided, or entirely ceased. An active ptyalism is, however, not unfrequently known to appear, when the tumour is about subsiding, and becoming soft and less painful. Both parotids may be similarly affected at one and the same time; but the commencement of disease in each is never simultaneous: several hours always intervene between the periods when inflammation is developed in the glands of the opposite sides.

*Rationale Symptomatum.* If we examine attentively the symptoms which our partial observations have enabled us to enumerate, we shall find cerebral derangement one of the earliest and most lasting. This, (though probably owing, in some slight measure, to the peculiar state under which the system is labouring at the moment of attack, and during its continuance,) is no doubt chiefly caused by the direct effects of the accumulation of retarded blood, upon the substance of the brain and its meninges. The natural consequence of the rapid enlargement of the tumour is, to obstruct the free circulation of the blood in the cervical vessels, by its mechanical pressure; but the supply through the carotid arteries, is equally impeded with the exit of fluid through the jugular veins. Of course, we are obliged at once to seek further for the explanation of this symptom of engorgement; and we find it readily by adverting to the fact, that while the removal of superfluous blood by the jugular veins is much obstructed, or almost cut off by the tumour, the brain is receiving a full supply through the vertebral arteries, whose long canal protects them from any injurious impression. The brain, therefore, is subjected to violent oppressions, by the superabundant and unnatural quantity of the nutrient fluid, and, as a reasonable inference, its functions become disordered, and its energy enervated. *Deafness*, such as is attendant upon this complaint, may be produced, either by the direct effects of pressure upon the Eustachian tube, or by the paralysis of the nervous system, originating in cerebral derangement. The malcondition of the nervous system will also readily account for the *cessation of pain* on the arrival of the tumescence at its acme.—The explanation of the alteration of the voice; the sense of strangulation; the difficulty of deglutition, must all be sought for in the mechanical effects of pressure. But why does ptyalism so frequently occur towards the termination of parotitis mercurialis, especially when the disease is likely to assume a favourable issue? To answer this question correctly, we must refer to the state of the general system at the commencement of diseased action. We have distinctly stated in our exposition of symptoms, that parotitis mercurialis *never* originates during the progress of a ptyalism; and that a mercurialized condition of the body is absolutely and indispensably necessary for its existence. As far as the scope of our observation has extended, we know of no single case, unless as connected with, or consequent upon pyrexia. Now, from the well known and distinct effects of a febrile diathesis, and of a mercurialized state of the system, upon the exhalent orifices of the secretory vessels; and from the generally received Hunterian principle, that two diseased actions of a general character, cannot co-exist at one and the same time, in the same person, we can readily perceive why ptyalism is not a symptom of the inflammation in question. In this disease, pyrexia is always present, previous to its acme, either in a general or a local form. Mercury may be exerting its specific influence, in the latter case particularly, upon other glands, while the parotid itself shows not the slightest evidence of its action; but the moment the local phlogosis subsides, it then appears.

*Diagnosis.* Fortunately for us, Parotitis Mercurialis can scarcely be confounded with any other affection of the same gland. Let this one rule be invariably borne in mind, and we can never be mistaken, that it *never* occurs as a primary disease, nor in any case can possibly exist where the system has not been under the influence of mercury in some form or other, for the cure of the previous malady. The suddenness of the attack, the extraordinary rapidity of its progress, the excessive degree of the inflammation, the acute lancinating pains by which it is attended, the general constitutional derangement, &c., all serve to point out its real and dangerous character, in contradistinction to cynanche parotidea; and the cold abscesses of the French, the critical abscesses of fevers; the only two diseases with which it can possibly be confounded, by the most Bœtian stupidity.

*Prognosis.* The prognosis in this diseese, is most generally unfavorable. If 24 hours have elapsed from the period of attack, until the application of remedial agents, the condition of the patient is highly critical. Every moment of delay adds to the certainty of death. If suppuration ensues, or the collection of purulent matter cannot be induced to evacuate externally, the case is almost hopeless, and the physician need hardly resort to any other than euthanasial remedies, to soothe the sufferer's last hours of agony and distress. The occurrence of a ptyalism is always favorable. The ratio of deaths from this disease, to recoveries, is, as far as we can judge, as that of four to one.

*Treatment.* In order more plainly to elucidate our therapeia, the medical treatment may be considered under three leading indications: 1st. of subduing the general pyrexial excitement when present; 2d., of procuring the speedy resolution of the tumour; and 3d., of supporting the system during collapse.

Of the 1st: general pyrexial excitement is not always attendant upon parotitis mercurialis; but sometimes it is present, and demands remedial attention. When this is the case, the usual antiphlogistic remedies must be resorted to at once. Venesection, prompt, and decisive, and carried to the extent of making a powerful impression on the system, and followed by active purgatives of a drastic nature, form the principal sanatory agents. If general nervous irritation become very violent, opiates, such as Pulv. Ipec. C., belladonna, hyoscyamus, acet. morph., &c., may be employed with some advantage. But it is to the 2d indication, that we are to turn all our attention and discrimination. We have already stated that the disease is peculiarly rapid in its progress; and proportionate to the severity of the inflammation, is the speed with which it terminates in its highly morbid character. No time should be lost—for every hour adds to the certainty of its fatal issue. So short, indeed, is the period for the employment of therapeutic agents, and in which the disease may be considered as under the control of medicine, that the delay even of a few hours may render abortive our most potent drugs, and seal the doom of the wretched victim. When, therefore, we are called to a case of this kind, our first resort should be (after the administration of anti-phlogistic means, if necessary,) the application of blister, covering the whole of the inflamed surface. As the surface of the affected parts is generally extremely insensible, it would be advisable to premise its application by some stimulent article, to excite the parts, such as hartshorn, decoct. of canthar. in spts., tereb., aq. ammon., or even in very desperate cases, nitric acid. It is only, however, in the first 36 or 48 hours, that epispastics can be of any real service. If they fail to have the proper effect on the first application, they should be frequently renewed, and assisted by the use of leeches. Should we fail in procuring the resolution of the tumour by this practice, and suppuration becomes unavoidable, or should we, unfortunately for the patient and ourselves, be called in just as this is commencing, we must endeavour, by every tangible means, to solicit it to the external surface, and prevent that most deplorable of all issues, the evacuation of the abscess internally. For this purpose, warm emollient poultices, renewed before they cease to return their warmth, should be constantly and immediately employed. The tumour in this situation is to be closely watched; and should there be, or should there not be a pointing of the abscess, we ought to open it at once, with the caustic or the lancet, after the lapse of a few hours, preferring the *hazard* of death by debility and exhaustion, to the *certainty* of it by suffocation, should the abscess burst inwardly.

During the formation and continuance of this suppurative and gangrenous stage, it is, that our 3d indication must be imperatively and rigidly followed. To preserve the system from sinking beneath the exhaustion and collapse, we must support it by the employment of stimulants and tonics, both of a medicinal and dietetic character.

Throughout the whole course of this disorder, the bowels are in a constipated condition, and it is indispensably necessary to correct this. For effecting this object, we know of no better agent than the following:—℞. sulph. sublim. oz. ss. Pot. sup. tart. dr. vi. ij.—one third of this to be given every hour, until purgation is attained. It should be employed every day.

Should the patient fortunately survive, the abscess after sloughing, is to be treated as a common abscess of any other part.

### Cases.

*Case I.* Mrs. B——— had been labouring under an attack of bilious remittent fever, and to all appearance was gradually recovering her health, under the alterative use of sub. mer. hyd., when she suddenly complained of a soreness and swelling under the right ear, in the region of the parotid gland, attended with a slight degree of pyrexia, and stiffness of the lower jaw. None of the specific effects of mercury were at that time perceptible. Unaware of the nature of the disease, (for it was our first case,) the usual discutient and emollient remedies were applied; but the tumour, instead of yielding, rapidly progressed, until, after running the course of many of the symptoms before described, it terminated about the third day in an external rupture of the abscess. The discharge of pus was inconsiderable, and the cerebral disorder much aggravated. Large quantities of the cellular tissue, of the surrounding muscular structure, and finally, of a substance resembling the parenchyma of the parotid gland, came away by the spontaneous action of the vessels, and exhibited a horrible ulcer, occupying the region of the angle of the jaw, larger than a Spanish dollar, through which the interior of the mouth was visible. The system sank rapidly beneath the irritative fever, until the fifth day put an end at once to her disease and sufferings. It may not be inappropriate for the remarks we shall hereafter advance, to mention, that during the whole of her confinement, she occupied a large and airy chamber, and that her bed was placed near a spacious window, thro' which currents of air had free access to the affected side.

*Case II.* Was communicated by my friend, Dr. Atlee. A convict, in the prison of his county, on the 13th day from the commencement of an ordinary remittent fever, was seized with a painful swelling in the seat of the parotid gland, about the size of an English walnut. Mercury had been used extensively, but the gums were healthy, and no signs of ptyalism. On the fourth day from this swelling, the other parotid was similarly affected. The common course of stimulants, such as vol. lin., &c., and even epispastics, aided by powerful purgatives, antimonials, and the use of the scarificator and cup was resorted to; but all in vain. The pulse increased in frequency, as did also the comatose symptoms in violence, until finally, the 7th day closed the patient's life. During the period of his febrile disorder, he was exposed to constant currents of cool air; and instead of adhering to the prescribed regimen, his access to, and use of cold water, was uninterrupted.

*Case III.* From the same source as the last, Susan C———, after a severe attack of bilious fever, which was fast yielding to medical treatment, on the 11th day of her illness, applied to her physician for advice respecting a painful swelling, which had appeared the night before, under her right ear, attended with considerable soreness upon pressure, and a frequent, contracted and small pulse. It had already attained the size of a ball two inches in diameter. A blister was immediately applied over the whole tumefaction, and a purge of sulph. sub. and pot. sup. tart. in large doses, repeated every 3 or 4 hours. On the subsequent day, it was found that the blister had not drawn well, but that the swelling had advanced but little. Another was re-applied, and the attendant symptoms of general irritation were corrected. This blister vesicated well; and on the following day, the tumefaction was checked entirely; the general and local irritation much relieved, and the excessive pain greatly abated. A tumour of the same nature was now discovered on the opposite side, and the same treatment of vesicants and purgation dispersed it by the next day. On the 6th day from the appearance of the swelling, a pointing, and some fluctuation was perceptible in it, and an emollient poultice was ordered: the next day the abscess was opened, and a small discharge of sero-purulent matter took place. On this day, the patient complained of soreness of the gums, and a ptyalism was, for the first time, observable. The poultices were continued: the strength supported by Cort Peruv. pulv., ter in die, and irritation allayed by Pulv. Ipecac. C. Sloughings subsequently occurred, and the ulcer was treated as an ordinary sore. It healed kindly, and in a few days the patient so far recovered her health, as to visit her friends. In this case, during the fever, cold water was constantly employed to relieve thirst; and she was also exposed to the damp air of a water-course.

*Case IV.* The subject of this, Lewis S———, a young man of 22 years, was slowly recovering from a long and protracted attack of bilious remittent, in which calomel had been freely administered, without producing its specific effect. He occupied a large room of a house long untenanted, and his bed was placed in such a position, that the air from the door was continually acting upon him; and his drinks were generally cold. When he was thought beyond all reach of danger, he was suddenly seized with a pain and soreness under the left ear, which soon became excruciating. When we first saw the tumour, it had attained a considerable size, and had involved all that side of the face in a general inflammation. The skin was extremely tense, communicated a hot, pricking sensation to the hand—was of a glossy and somewhat purple redness, and the whole system was labouring under severe irritation. As the nature of the complaint was too obvious to be mistaken, we resorted at once to vesicants, premised by stimulant embrocations; and to the constant use of purgatives of sulph. sublim., and pot. sup. tart. in large doses, every hour; with an opiate to allay irritation. In the course of 12 or 15 hours, the pain began to abate, and the swelling to decrease. The blisters were dressed with unguent basil., to promote suppuration. After a few days the tumour disappeared entirely, and the patient soon regained his health. This was a marked case of the best of all terminations, that of resolution.

We might proceed to illustrate this disease with some three or four more cases, but the leading features of all were similar to those already described, though the terminations of all, owing to inexperience, were fatal.

*Causes.* From an attentive perusal of what we have here laid down, and from our cases, it must be evident that there are in this disease, two active and predisposing causes, mercury, and fever, or some other previous general affection. It is not necessary for us to enter into any prolix discussion of the respective effects of each upon the system—they are well known and generally understood. Suffice it to say, that we have in them two powerful agents, both causing a morbid impressibility, though acting in opposite directions in its production. They cannot exist at the same time; and which ever of the two prostrates the other, the debilitating and irritating consequences of the one, will be heightened by the present or previous effects of the other. Yet, unless the relaxing and disgorging power (not the specific effect,) of mercury prevail, we believe that the disease in question can never occur. To explain: "If mercury is exhibited in fever, so as to produce its *specific* constitutional effect, agreeably to the law of *unities*, the fever must yield; but if this point is not attained, it will, in some measure, still continue. In the first case, the vanquished fever will have added to the susceptibility of the frame; and in the latter, the mercurialized state of the body will operate in the same manner." Thus it is, then, that these two antagonising powers, acting upon a syncratistical principle, lay the body open to the action of morbific agents, from without. It is in this condition of the system, that the *exciting* or *proximate* cause, *cold*, acts with such terrible energy. In every case we have adduced, and every one we have seen, *cold*, either thro' an aqueous or aerial medium, had acted upon the part. We need enter into no laboured effort to prove, that as cold is a powerful remedy in the removal of diseases, it must, of necessity, be equally efficient in producing them. One thing, in our mind at least, is certain, and that is, that local inflammations owe their origin to this agent, far more frequently than to any other. In relation to the subject before us, we are fully convinced, from our own limited experience, that *cold*, in one or other of its forms, acts as the proximate and exciting cause in the production of Parotitis Mercurialis.

In closing our remarks upon this interesting disorder, we will enter into the following recapitulation: 1st. that parotitis mercurialis never occurs as a primary, idiopathic disease; but, perhaps, in every case after a fever: that a mercurialized condition of the system is essentially requisite for its existence; and that cold is an indispensible agent in its production. 2d. That its progress is extremely rapid: its curable condition is very limited; and that the prognosis is generally unfavorable. 3d. That the usual discutients are useless, and worse than useless, as they waste valuable time: that the only safe reliance is upon the course we have prescribed; and that the tumour should always be opened in preference to running the hazard of internal rupture.

We cannot take leave of our subject without expressing an ardent solicitude, that so peculiar a disease may be more closely investigated by men of superior talents, and longer experience.

*Note.* Dr. Morton, in a letter to the editor, assigns the credit of the principal part of this essay, to the inaugural thesis of Dr. Atlee; but as the latter gentleman was unwilling to publish his thesis, we have taken upon ourselves the responsibility of publishing it under its present form. Cases of this kind, we think, are unfrequent: but we have met with one or two, somewhat analogous, under circumstances very similar. They occurred in old and debilitated habits, at the close of an attack of bilious fever, in which calomel had been administered, not very liberally, but to a greater extent than we would again be willing to give it under similar circumstances. Very slight salivation ensued, which was suddenly arrested, and the tumour quickly appeared, excessively painful, and very tedious, but at length terminating favourably, by healthy suppuration. Some unfavorable remarks were made, though we thought unjustly, as we considered the affection owing to some idiosyncrasy, which we could neither foresee nor control,—the calomel, probably, from some accidental circumstance, having affected the parenchyma of the gland, instead of its secreting vessels. We suspected at the time, that the too early use of the quinine, by introducing a slight continued form of febrile irritation, with a red, dry tongue, had some influence in producing this result. The course pursued was, bitters, aloes and rhubarb, as a laxative, fomentations and poultices to the inflamed gland. F.

